# Integrating palliative care into neurology services: what do the professionals say?

**DOI:** 10.1136/bmjspcare-2017-001354

**Published:** 2017-08-03

**Authors:** Nilay Hepgul, Wei Gao, Catherine J Evans, Diana Jackson, Liesbeth M van Vliet, Anthony Byrne, Vincent Crosby, Karen E Groves, Fiona Lindsay, Irene J Higginson

**Affiliations:** 1 Department of Palliative Care, Policy and Rehabilitation, Cicely Saunders Institute, King’s College London, London, UK; 2 Department of Palliative Medicine, Sussex Community NHS Foundation Trust, Brighton, UK; 3 Department of Palliative Medicine, Cardiff & Vale University Health Board, Cardiff, UK; 4 Department of Palliative Medicine, Nottingham University Hospitals NHS Foundation Trust, Nottingham, UK; 5 Queenscourt Hospice, Liverpool, UK

**Keywords:** chronic conditions, communication, clinical decisions, neurological conditions

## Abstract

**Objectives:**

Evaluations of new services for palliative care in non-cancer conditions are few. OPTCARE Neuro is a multicentre trial evaluating the effectiveness of short-term integrated palliative care (SIPC) for progressive long-term neurological conditions. Here, we present survey results describing the current levels of collaboration between neurology and palliative care services and exploring the views of professionals towards the new SIPC service.

**Methods:**

Neurology and palliative care teams from six UK trial sites (London, Nottingham, Liverpool, Cardiff, Brighton and Chertsey) were approached via email to complete an online survey. The survey was launched in July 2015 and consisted of multiple choice or open comment questions with responses collected using online forms.

**Results:**

33 neurology and 26 palliative care professionals responded. Collaborations between the two specialties were reported as being ‘good/excellent’ by 36% of neurology and by 58% of palliative care professionals. However, nearly half (45%) of neurology compared with only 12% of palliative care professionals rated current levels as being ‘poor/none’. Both professional groups felt that the new SIPC service would influence future collaborations for the better. However, they identified a number of barriers for the new SIPC service such as resources and clinician awareness.

**Conclusions:**

Our results demonstrate the opportunity to increase collaboration between neurology and palliative care services for people with progressive neurological conditions, and the acceptability of SIPC as a model to support this.

**Trial registration number:**

ISRCTN18337380; Pre-results.

## Background

Palliative care has been proposed to help meet the needs of patients with progressive non-cancer conditions such as long-term neurological conditions (LTNCs).^1 2^ However, there is little or no evidence on the best ways of providing palliative care for these patients. Should it be at the ‘end of life’ or earlier such as at the point of diagnosis? Our own phase II randomised controlled trial (RCT) in patients with multiple sclerosis (MS) showed a reduction in symptoms and caregiving burden, following short-term integrated palliative care (SIPC) compared with standard care.^3^ More recently, a pilot RCT in Italy evaluating the impact of a new specialist palliative care service for patients with a range of LTNCs found significant improvements in quality of life and physical symptoms.^4^ Whether more people living with different LTNCs can benefit from SIPC and whether it can be routinely used in practice to improve care quality are of interest.

OPTCARE Neuro is a multicentre RCT evaluating the effectiveness of SIPC for progressive LTNCs (ISRCTN18337380). The SIPC service being trialled is defined as three palliative care visits over 6–8 weeks. This is a phase III RCT in patients with a range of LTNCs including: MS, motor neuron disease (MND), idiopathic Parkinson’s disease, progressive supranuclear palsy and multiple system atrophy. The overall aim of OPTCARE Neuro is to evaluate the clinical and cost-effectiveness of SIPC to optimise care for people with LTNCs. In addition to understanding the effectiveness of this service, it is also important to understand and be aware of current service provisions and the views of professionals involved in providing care for this patient group. The complexity of delivering and evaluating palliative care services requires the accumulation of knowledge from multiple sources and will depend on interprofessional behaviours.^5 6^ It is therefore valuable to explore clinicians’ views and opinions when shaping emerging services and informing future requirements. With that in mind, we conducted a short online survey with neurology and palliative care professionals. The main aims of the survey were to:

- understand what current levels of collaboration exist between the two specialties;
- explore the expectations and views of clinicians towards the SIPC service being trialled.

## Methods

Research teams at six UK trial centres (London, Nottingham, Liverpool, Cardiff, Brighton and Chertsey) identified local neurology and palliative care professionals who were then approached via email by the central trial team. Professionals were informed that by completing the survey, they provided informed consent for use of their anonymised data. The surveys consisted of multiple choice or open comment questions, 13 (for neurology) or 10 (for palliative care) with responses collected using online forms. The survey was launched in July 2015 and closed April 2016. The study was approved by the National Research Ethics Service Committee London South East (REC number: 14/LO/1765).

## Results

**Figure 1:**
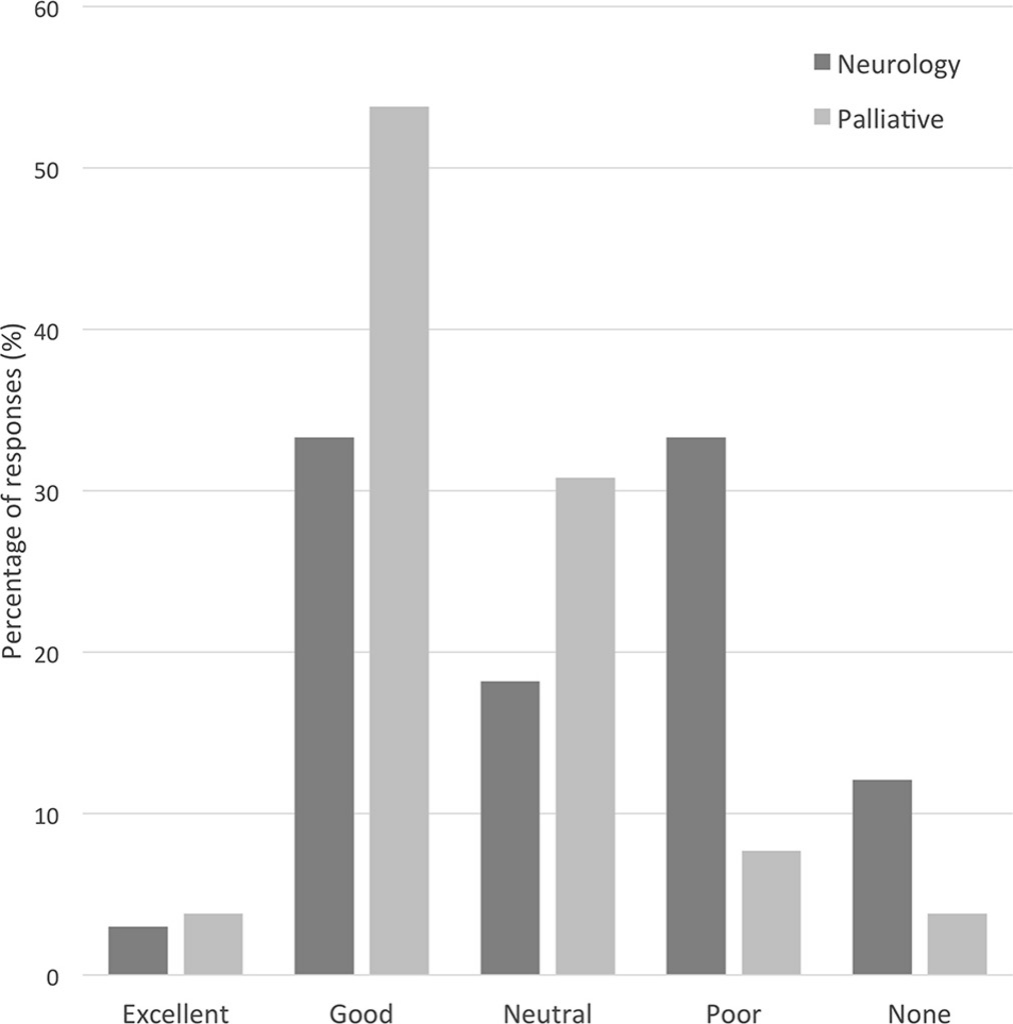
Neurology and palliative care professionals’ ratings of their current levels of collaboration with the other specialty.

The survey received responses from 33 neurology and 26 palliative care professionals (20% response rate). Two-thirds of respondents in both groups had over 10 years of experience in their respective fields. Current levels of collaboration between the two specialties were reported as being ‘good/excellent’ by 36% of neurology professionals and by 58% of palliative care professionals. However, nearly half (45%) of neurology compared with only 12% of palliative care professionals rated current levels as being ‘poor/none’ (see figure 1). When asked if there were any particular disease areas where links were better, both groups reported stronger links for MND. In addition, both professional groups felt that the new SIPC service being trialled would influence future collaborations for the better (65%–70% in both groups).

Participants were also asked what they thought would be the main barriers for the new SIPC service (see table 1). The most commonly identified barriers by neurologists were resources, clinician awareness of services offered, continuing collaborations and communication between teams beyond the trial and geographical limitations. Similarly, palliative care professionals also identified resources and clinician awareness (and, importantly, the appropriateness of referrals they may receive) as barriers. However, the key barrier they identified was that there may be a possible need for longer-term care beyond that offered by the SIPC service. They also drew attention to patient perceptions of palliative care as a potential barrier.

**Table 1:** Barriers identified by professionals

Neurology professionals	
Resources	“Workload volume”
	“Increasing number of referrals will put the service to the same problem as in other healthcare foci - waiting times and availability may fall behind which happens to all services sooner or later. Sadly, the better services the sooner you will use your resources”
	“Resources and how best to communicate with varied services/provision”
	“Social care funding. Pent up demand”
	“Cost”
Clinician awareness and acceptance	“Knowing what services are available in localities”
	“Awareness, especially in primary care”
	“Clinician awareness”
	“Senior medics previous practices”
	“Getting themselves trusted by the consultants”
Continuing collaborations and communication	“Creating good links between the MS team and the palliative care team”
	“Communication issues between different care providers”
	“Ongoing joined-up work to ensure care continuity and no repetition of service provision”
	“Establishing a robust and efficient process for communicating with all disciplines involved in the patient’s care …”
Geography	“Geographical limitations - a lot of our patients are not local”
	“Geographic’s”
	“Many of our patients live a long way from the centre to allow active engagement”
Patient perceptions and acceptance	“Patient resistance”
	“The term hospice which often patients and families feel has a strong association with immanency of dying. People often express fear of contact with a hospice if they do not feel that they are close to death”

Palliative care professionals	
Longer-term service provision	“Recognising the need but not being able to back it up with longer-term intervention”
	“Short-term intervention and longer-term follow-up and review may be needed”
	“Discharging patients after a short intervention, particularly if the service has made a difference to patient and families”
	“Discharge at the end of the intervention may be difficult as the patient has benefited from the intervention and may be reluctant to stop it. This may be particularly difficult also for carers…”
	“Ending the contact after specified time as I think we are generally not very good in SPC at discharging patients/terminating our involvement…”
	“Time constraints - therapy intervention cannot always be addressed and completed within 6–8 weeks and require longer-term follow-up…”
Resources	“Workforce concerns for current team delivering SIPC”
	“If successful, there would be funding/capacity issues if the service was to continue”
	“Time, money”
	“Time pressures especially if majority want home visits”
	“Time and resources”
Clinician awareness and acceptance	“Lack of understanding what can be done. Not appreciating the importance for regular review”
	“Recognition of need for palliative care”
	“Making sure all teams aware how to access service & aware of its role”
	“I think there is a mismatch between what neurology think palliative care can offer and what we think we can offer…”
Patient perceptions and acceptance	“Patient and carer perceptions of palliative care and the role of specialist palliative care teams”
	“Reluctance to attend due to preconceptions of hospice being only for people to die in rather than seeing us as a team to help manage symptoms and discharge back to community care - with ongoing support as necessary”
	“Patients who feel they have already tried everything and are fairly rigid in their approach to trying different ways of doing things, for many versed and valid reasons”

MS, multiple sclerosis; SIPC, short-term integrated palliative care; SPC specialist palliative care.

## Discussion

Our results demonstrate that collaborations can be improved and both specialties are positive about the impact the new SIPC service will make. However, the barriers identified highlight areas for consideration and further exploration. Patient perceptions of palliative care was identified as a potential barrier to the successful integration of neurology and palliative care services. It is equally important for neurology professionals to have the right understanding of palliative care and to recognise the potential benefit of palliative care for their patients. Indeed, previous studies have demonstrated that the topic of palliative care can still often lead to anxiety in patients, caregivers as well as healthcare professionals.^7 8^ There is an emphasis on the need for integrated working along with improved education and awareness in order to make palliative care more recognised and more accessible for non-cancer conditions such as LTNCs.^9 10^ As reported by both specialties, resources must be carefully considered and systems developed for calling on palliative care specialists when truly necessary. The small number of respondents highlights the challenges of conducting research among busy health professionals; however, the geographical variation is an advantage of the survey.

## Conclusions

Our results demonstrate the opportunity to increase collaboration between neurology and palliative care services for people with progressive neurological conditions, and the acceptability of SIPC as a model to support this. This survey will be repeated at the end of the trial to understand how collaborations and views have changed, whether the SIPC service has affected the care process and to identify areas for improvement. These survey results will be integrated with the qualitative trial findings to provide a wider context about the effects of SIPC on the processes of care, and the ways in which it might be working effectively.
